# Editorial: Interferon-λs: New Regulators of Inflammatory Processes

**DOI:** 10.3389/fimmu.2019.02117

**Published:** 2019-09-04

**Authors:** Ivan Zanoni, Charlotte Odendall

**Affiliations:** ^1^Division of Immunology, Division of Gastroenterology, Boston Children's Hospital, Boston, MA, United States; ^2^Department of Infectious Diseases, School of Immunology and Microbial Sciences, King's College London, London, United Kingdom

**Keywords:** interferon, type III IFNs, barrier, inflammation, IFN lambda

Interferons (IFN) were the first family of cytokines to be discovered, with type I IFNs in 1957 followed closely by type II IFN in 1965. Type I IFNs are a large family comprised of IFNαs, β, and other subtypes while IFNγ is the sole type II family member. Our understanding of IFN function was binary for a long time, with type I IFNs considered mainly antiviral while IFNγ was classified as the antibacterial IFN. It isn't until 2003 that a third family emerged, type III IFNs also known as IFNλ1-4 or IL29, Il28A-C. Type III IFNs are functionally closer to type I IFNs as they have potent antiviral functions and induce a largely overlapping family of interferon stimulated genes (ISGs). These functions are best described in epithelial cells, the primary target of type III IFNs, but more recent immunomodulatory roles of type III IFNs are emerging. This topic discusses several aspects of type III IFN biology, from its similarities and differences with type I IFNs, to functions in different tissues and models.

## Antiviral Activities of type III IFNs at Mucosal Sites

One of the distinct features of type III IFN biology is the restricted expression of its receptor. The IFNλ receptor (IFNLR) is composed of two chains including a unique chain, IL28Rα (IFNLR1), whose expression is most predominant on cells of epithelial lineage. As a consequence, the function of type III IFNs are most important at mucosal surfaces and type III IFNs have been emerging as critical regulators of immunity at barrier sites. In this topic Hemann et al. deliver a very nice overview of the regulation of IFNλ gene expression in response to viral infections. This review also compares the different functions of type I and III IFNs in different models. Lee and Baldridge dives deeper in the antiviral functions of IFNλs in the intestine. Both these review articles include comprehensive tables outlining the functions of IFNλs in response to different viral challenges. Andreakos et al. take a closer look at the roles of type III IFNs in the respiratory tract. IFNλs are “front-line guardians” and contribute to immunity against acute viral infections but also chronic respiratory diseases. This theme is also tackled by Sopel et al. that specially discuss the role of IFNλ in asthma.

## Differences Between type I and III IFN Signaling in Polarized Epithelial Cells

The antiviral state activated by type I and III IFNs is mediated by the induction of a large family of ISGs. In contrast to IFNγ that induces genes regulated by gamma activated sequence (GAS) promoters, type I and III IFNs both induce genes containing IFN stimulated response elements (ISRE). The ISGs induced by type I and III IFNs were therefore considered to be largely identical. Here, Selvakumar et al. use a mouse polarized intestinal epithelial cell line to identify a subset of ISGs uniquely induced by IFNλ2. Interestingly these genes are only strongly induced in polarized gut epithelial cells, but not in unpolarized cells, bone-marrow derived dendritic cells or primary lung epithelial cells. Polarization of intestinal epithelial cells increased expression of both chains of the IFNLR, while the type I IFN receptor (IFNAR) chains remained unchanged.

In a parallel study Bhushal et al. compare the strength and frequency of ISG expression upon type I and III IFN treatment. They show that almost all unpolarized cells in culture respond to high concentrations of IFNβ while IFNλ responsiveness plateaus below 50% of cells. Histone deacetylase inhibitors (HDAC) restore frequency of responsiveness upon IFNλ treatment revealing the role of epigenetic regulatory mechanisms in ISG expression downstream of type III but not type I IFN stimulation. As seen above, upon cell polarization, full IFNλ responsiveness was restored, but this response was no longer enhanced by HDAC inhibition. It would be interesting to investigate if the expression IFNλ-specific ISGs identified by Selvakumar et al. is particularly regulated by epigenetic modifications.

In another study investigating signaling downstream of type I and III IFNs in intestinal epithelial cells, Pervolaraki et al. use human mini-gut organoids and polarized human cell lines. They find that both IFN classes induce ISGs and control virus infection but that they do so via separate pathways. In addition to STAT1, 2, and 3, both IFNs phosphorylated MAP kinases (MAPKs). However, inhibition of MAPKs only affected the ability of IFNλs to control viral infection. Interestingly this effect was independent of STAT1 phosphorylation showing that the two pathways are probably independent. Whether these findings are linked to the differential ISG regulation observed in the previously mentioned studies will need to be addressed in the future.

## Immunomodulatory Roles of Type III IFNs

As discussed in most papers in this topic, in contrast to IFNAR which is ubiquitously expressed, IFNLR expression is limited to a small number of lineages. It is widely accepted that IFNLR is most strongly expressed on cells of epithelial origin. Recently, neutrophils also appeared as target cells, while there is some debate as to natural killer (NK) cells or dendritic cells (DCs) were IFNλ-responsive. In this topic, Zanoni et al. review the roles of IFNλs on immune cells. IFNλs function on neutrophils to halt their migratory capabilities, or inhibit reactive oxygen species production and neutrophil extracellular trap release. They also discuss that while type III IFNs do affect the functions of NK cells, it is most likely indirect as there is limited evidence that NK cells express the IFNLR or are IFNλ responsive. Conversely, conventional mouse DCs and human plasmacytoid dendritic cells (pDCs) do respond to type III IFN stimulation. IFNλ treatment of human pDCs leads to JAK/STAT activation and induction of ISGs, as well as up regulation of certain cytokines and surface markers, and increased survival. For a deeper look, the different functions of IFNλs on pDCs is synthesized by Finotti et al. in this topic.

## Treatment Options With Type III IFNs

Type I IFN are an accepted treatment strategy for a number of inflammatory or infectious diseases including viral hepatitis. However, the efficacy of these treatments is not optimal and they come with many debilitating side effects. The emergence of type III IFNs as potent antiviral cytokines opened new treatment options for several afflictions including patients with chronic viral infections. In this topic, Phillips et al. report a clinical trial using pegylated IFNλ in chronic hepatitis B (CHB) patients. They find that in combination with directly acting antiviral therapy, peg-IFNλ treatment increased antiviral cytokines in the serum, and enhanced NK cell function while maintaining HBV-specific CD8+ T cell functions. Overall a better control of viral replication was observed.

## Conclusion

Type III IFNs were identified over 17 years ago and are almost reaching majority. While work in the infant years of IFNλs mostly listed the similarities between the type I and III IFN systems, more recent work has revealed important differences. Type III IFNs have specific functions on epithelial and immune cells that make them key actors in immunity and regulators of inflammation at mucosal sites. As IFNλs continue to attract more interest from infection biologists and immunologists, many more important discoveries can be expected about this fascinating family of cytokines.

## Author Contributions

CO and IZ wrote the manuscript.

### Conflict of Interest Statement

The authors declare that the research was conducted in the absence of any commercial or financial relationships that could be construed as a potential conflict of interest.

